# Object Weight and Hand Dominance Impact Kinematics in a Functional Reach-to-Drink Task in School-Aged Children

**DOI:** 10.3390/s24165421

**Published:** 2024-08-22

**Authors:** Julia Mazzarella, Daniel Richie, Ajit M. W. Chaudhari, Xueliang Pan, Eloisa Tudella, Colleen K. Spees, Jill C. Heathcock

**Affiliations:** 1 School of Health and Rehabilitation Sciences, College of Medicine, The Ohio State University, Columbus, OH 43210, USA; 2 Department of Biomedical Engineering, College of Engineering, The Ohio State University, Columbus, OH 43210, USA; 3 Center for Biostatistics, Department of Biomedical Informatics, College of Medicine, The Ohio State University, Columbus, OH 43210, USA; 4 Departamento de Fisioterapia, Universidade Federal de São Carlos, São Carlos 13565-905, SP, Brazil

**Keywords:** children, coordination, early childhood, kinematics, motion analysis, pediatrics

## Abstract

This study evaluates the effects of object weight and hand dominance on the end-point kinematics of the hand-to-mouth (withdrawal) movement in a functional reach-to-drink task for typically developing school-aged children. Using 3D motion capture, speed (average velocity and peak velocity), straightness (ratio), and smoothness (number of velocity peaks and log dimensionless jerk) of hand movements were calculated for the withdrawal motion with three different bottle weights (empty, half-filled, and full). Average velocity (550.4 ± 142.0 versus 512.1 ± 145.6 mm/s) and peak velocity (916.3 ± 234 versus 842.7 ± 198.4 mm/s) were significantly higher with the empty versus half-filled bottle and with the non-dominant (average: 543.5 ± 145.2 mm/s; peak: 896.5 ± 207 mm/s) versus dominant (average: 525.2 ± 40.7 mm/s; peak: 864.2 ± 209.2 mm/s) hand. There were no differences in straightness or smoothness. These findings indicate that increasing weight in reach-to-drink task puts greater constraints on the task. The slower movements with the dominant hand might denote better precision control than the non-dominant hand. The quantitative motion capture results show average values for the kinematic variables for a functional reach-to-drink task in a typically developing population of school-aged children with changing weights of the bottles that are relevant to a real-life scenario. These results could inform the design of individualized therapeutic interventions to improve functional upper-extremity use in children with neurodevelopmental motor disorders.

## 1. Introduction

Reaching and grasping are essential motor functions for school-aged children to complete activities of daily living (ADLs) such as eating, drinking, and dressing and to participate in school and recreation [1,2,3]. These skills are often challenging and a major focus for rehabilitation for children with neurodevelopmental motor disorders in their upper extremities, such as those with cerebral palsy (CP) [2,4,5,6,7,8,9,10,11,12,13,14]. Dynamic Systems Theory (DST) is the most prominent and accepted theory of motor development applied in rehabilitation. DST indicates that every reach and grasp performed throughout a child’s day is unique. Each reaching movement is the result of a complex interplay of the child’s motor control; the constraints of the task, such as initial and target locations of the object; and the characteristics of the object itself [15]. Examining changes in kinematics of functional reaching movements in typically developing children with various task constraints in the context of DST is necessary to inform targeted treatments for children with upper-extremity neurodevelopmental motor disorders. Much research has applied DST to understand reach development and laterality in infants and toddlers and inform early intervention practices for children with neurodevelopmental motor disorders [16,17,18]. There remains a lack of research in reaching behaviors and functional upper-extremity coordination in school-aged children [19]. This research is crucial to inform rehabilitation interventions for children with neurodevelopmental motor disorders as they continue to grow and develop. It is particularly necessary from a DST perspective to understand the interplay of task constraints such as object weight and use of the dominant versus non-dominant hand. Knowledge of how these constraints influence reaching patterns in children with typical development will inform assessment and intervention for children with upper-extremity neuromotor disorders.

**Figure 1:**
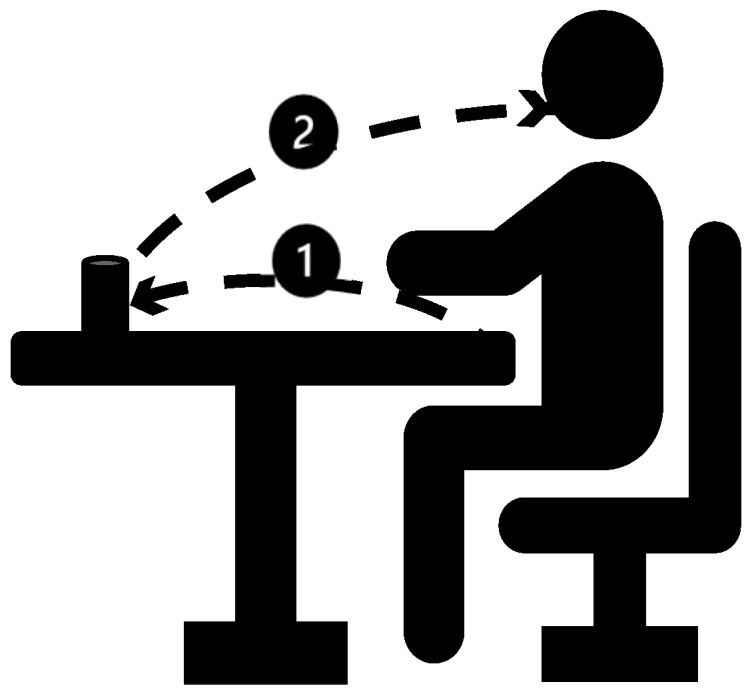
Depiction of the prehension (1) and withdrawal (2) movements in a reach-to-drink task.

Execution of a reach-to-grasp task requires a complex interplay of multiple systems in several distinct phases, each of which are kinematically different. Butler et al. [1] defined a “Reach and Grasp Cycle”, which describes the reaching and grasping phases of a reach-to-drink task. This cycle was adapted by Machado et al. (2019) to four movement phases: (1) reaching toward the object, (2) transporting the object to the mouth, (3) returning the object to the table, and (4) returning the hand to the table. Previous studies in adults have defined phase 1 as the prehension movement and phase 2 as the withdrawal movement (Figure 1) [20,21,22,23,24]. The prehension phase in a reach-to-drink task is visually guided and requires motor planning to aim, shape, and orient the hand to grasp the object in a manner that is most efficient to complete the subsequent phases of the task [23]. The withdrawal phase is guided by the somatosensory system and requires motor control to maintain the grip on the object and accurately aim to the mouth [23]. Because the various constraints of the task change from one instance to the next, such as the location and weight of the bottle, DST tells us that each instance of a reach-to-drink task could be kinematically different as the dynamic systems adapt. Due to damage to the brain systems that contribute to motor control and motor planning, children with neuromotor disorders have demonstrated deficits in various phases of a reach-to-drink task, the extent of which is dependent upon the location and severity of their brain injury [20,25,26].

Object constraints include object characteristics like size, shape, and weight, while task constraints include the location and orientation of the object. From previous studies in adults, we have some insight into how object and task constraints impact prehension movements. Both the task being performed and the object being grasped impact end-point kinematics of the prehension reach, selection of grasp, and hand orientation [21,22,27,28,29,30,31,32,33,34,35]. Because withdrawal movements are partly controlled by a different area of the brain, it is possible that the effects of object and task constraints could be different than for prehension movements, but this has yet to be investigated [27]. In a recent study, we explored the differences between a reach-to-drink and reach-to-eat task in typically developing school-aged children using the Reach and Grasp Cycle, and found significantly slower withdrawal movements in the reach-to-drink task compared to the reach-to-eat task [36]. This provides preliminary insight into how constraints impact the withdrawal movement but controls to identify the effects of individual constraints were lacking. Based on DST, the shape, weight, and size of the object as well as the differing goals of the tasks would have all influence the planning and control of the movement. The present study was designed to isolate the effects of object weight exclusively in a single reach-to-drink task. Weight is a characteristic that is easy to manipulate in a clinical setting, and the knowledge gained from this study will enable more precise design of individualized treatments for children with neuromotor disorders.

Dominant versus non-dominant or right versus left hand differences have also yet to be evaluated in the withdrawal phase of a reach-to-drink task in typically developing school-aged children. Prehension phase end-point kinematics have already been well documented. Differences in end-point kinematics between the dominant and non-dominant hands in the prehension phase of reaching emerge by age 3 in typically developing children and strengthen with development until a plateau at 8–11 years old, which is considered adult-like [37,38,39,40,41,42,43]. In children with neurodevelopmental motor disorders that impact one side of the body, such as hemiplegic CP, a hand preference for the unaffected side can emerge as early as 12 weeks [44,45,46,47,48,49]. In healthy adults, the dominant hand typically moves straighter and smoother in the prehension movement [35,50]. It is important to know the difference in kinematics between dominant and non-dominant hands in the withdrawal motion, as this could further inform differences in motor-impaired populations.

The aim of the current study was to identify the impact of object weight on upper-extremity timing and coordination of withdrawal movements in a functional, ADL reach-to-drink task in typically developing school-aged children for both the dominant and non-dominant hand. We calculated the speed (average velocity and peak velocity), straightness (straightness ratio), and smoothness (number of velocity peaks and log dimensionless jerk) of dominant and non-dominant hand movements in a functional reach-to-drink task with three different bottle weights based on liquid levels: empty, half, and full. Based on the previous literature on object constraints in adults, we hypothesized that with increasing bottle weight, hand movements would be slower, less straight (higher straightness ratio), and less smooth (more velocity peaks and more negative log dimensionless jerk) [21,22,27,28,29,30,31,32,33,34,35]. Based on previous comparisons of functional reach in children with typical development to children with CP (without regard to hand) as well as comparisons of the dominant versus non-dominant hand in adults, we hypothesized that the dominant hand would move faster, straighter, and smoother than the non-dominant hand [1,8,35,50]. Results from this study improve our understanding of how object and task constraints impact the kinematics of functional ADL reaching tasks in school-aged children in order to inform individualized treatment design for children with neuromotor disorders. 

## 2. Materials and Methods

### 2.1. Participants

Participants were a convenience sample of 33 children ages 8–11 years recruited from schools in low-resource neighborhoods in Columbus, OH, USA. Exclusion criteria included (1) being unable to follow instructions given in English and (2) any motor impairment preventing the child from being able to perform the reach and grasp tasks. Written informed consent was obtained from the parent or caregiver of each participant.

### 2.2. Procedure

All participants completed their assessment at the Pediatric Assessment and Rehabilitation (PEARL) Laboratory at The Ohio State University. Hand dominance was determined based on the child’s preferred writing hand. Children were outfitted with eleven 8 mm diameter retroreflective markers placed on their sternum, bilateral acromia, lateral epicondyles, radial and ulnar styloid processes, and heads of the third metacarpals. Markers were also placed on the four corners of the table, the back of the chair, and the bottles. Children were positioned in a chair so that their hips, knees, and ankles were flexed to 90°. The bottle was positioned in front of the child at 75% of their arm’s length [8,36,51]. This position was marked with tape for each participant. The bottles were transparent 12 oz juice bottles, which varied in weight per trial (Table 1). The three bottle weights were chosen to reflect the functional task of drinking, where the fluid level would decrease in the bottle as the individual takes more sips. Due to precautions taken during the COVID-19 pandemic, participants were required to wear masks, and bottles remained capped. Participants were asked to touch the bottle to their mask to simulate drinking. Simulations of functional tasks are common in the literature when measuring upper-extremity kinematics [52]. The participants began with their hands resting on the table at a marked position, shoulders neutral, elbows flexed to 90°, and wrists neutral. For each trial, participants were given the following instruction: “Reach for the bottle, pick it up, pretend to take a drink, return the bottle to the marked position on the table, and return your hand to the start position. Do this twice.” Participants were permitted to move their trunk freely but were asked to keep their feet flat on the floor. Participants were allowed one practice trial per hand with each bottle weight and then asked to complete two repetitions of the reach-to-drink activity for each bottle weight with each hand. The trial order was the same for each participant, starting with the empty bottle, then the half-filled bottle, then the full bottle. Participants started with their dominant hand, followed by the non-dominant. Movement was recorded by a 10-camera VICON Motion Capture system at 120 Hz and filtered at 4 Hz with a low-pass Butterworth filter.

### 2.3. Data Analysis

The withdrawal motion of bringing the bottle to the mouth was visually identified by three reliable coders and marked in the Vicon Nexus v2 processing software [53]. The coders were trained until they reached a criterion of 90% agreement on nine trials prior to coding individually. The beginning of the withdrawal movement was marked as the frame in which the hand–bottle dyad began to move toward the participant’s body. The end of the withdrawal movement was marked as the frame in which the bottle contacted the participant’s mask. A custom MATLAB script was used to calculate spatial and temporal variables of movement (Table 2) [54]. Several of these variables have previously shown good within- and between-day reliability in typically developing children performing the Reach and Grasp Cycle [1].

General linear mixed models for repeated measures were used to compare these five variables by bottle fill, hand, and bottle fill–hand interaction. The a priori alpha level was set to α = 0.05. Cohen’s d effect sizes were calculated for all effects. Post hoc testing was performed using Fisher’s least-significant difference (LSD) to compare bottle fill levels. The Bonferroni correction was applied to reduce the probability of Type I error, setting α = 0.017 for post hoc tests.

## 3. Results

### 3.1. Participant Characteristics

Two participants were excluded from the final analysis due to a data collection error. The final sample consisted of 20 female and 11 male, typically developing children. The mean age was 9.4 ± 0.8 years (*n* = 3 eight-year-olds, *n* = 14 nine-year-olds, *n* = 12 ten-year-olds, and *n* = 2 eleven-year-olds), and 5 were left-hand-dominant and 26 right-hand-dominant. There were no significant differences between right-hand- and left-hand-dominant individuals or between age groups in the sample when we included these variables in the model. Given the large differences in sample sizes between the age groups and left-hand- vs. right-hand-dominant groups, we chose to combine the groups for final analysis. Participant ethnicity was 13% Latinx, and race distribution was 39% white, 45% black, 6% Asian, and 10% biracial. Annual household income for participants’ families was USD 75,000 for 42%, between USD 50,000 and USD 74,999 for 16%, between USD 25,000 and USD 49,999 for 26%, and between USD 10,000 and USD 24,999 for 10%, and 6% chose not to respond to this question. 

### 3.2. Main Effects

**Figure 2:**
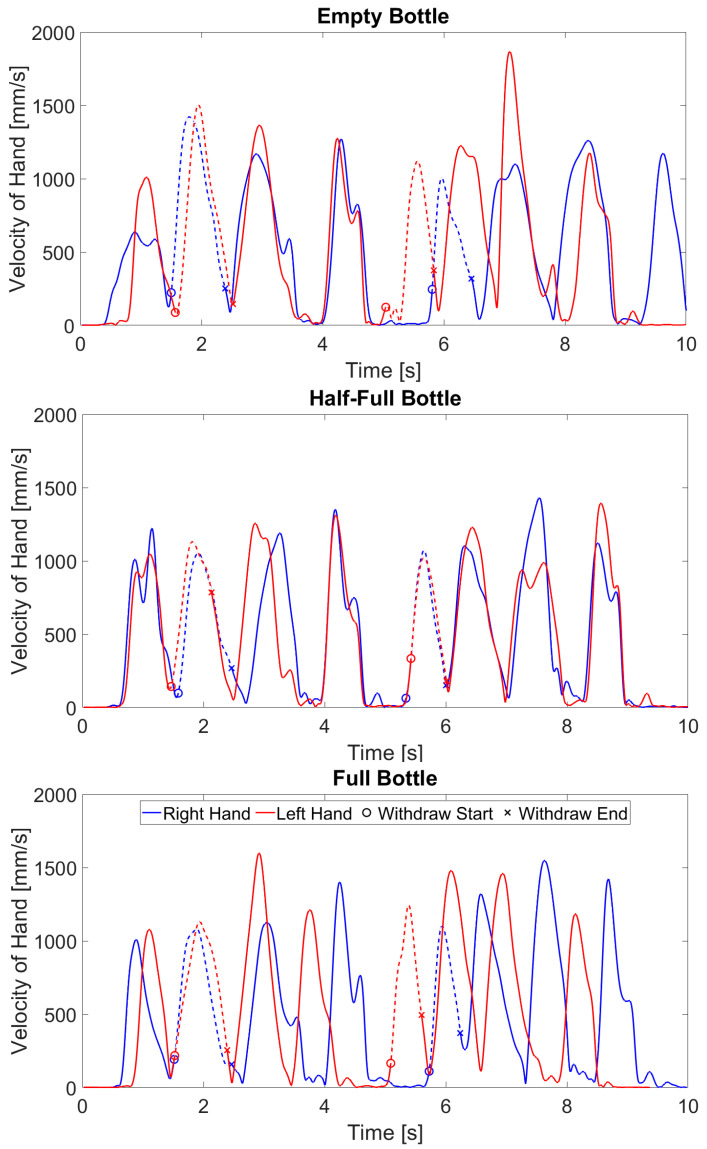
Sample velocity profiles for each condition.

**Figure 3:**
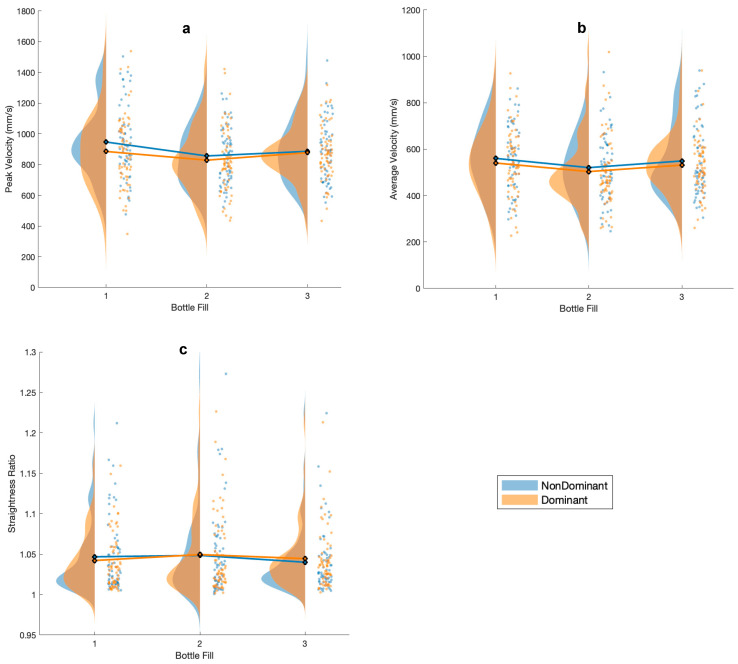

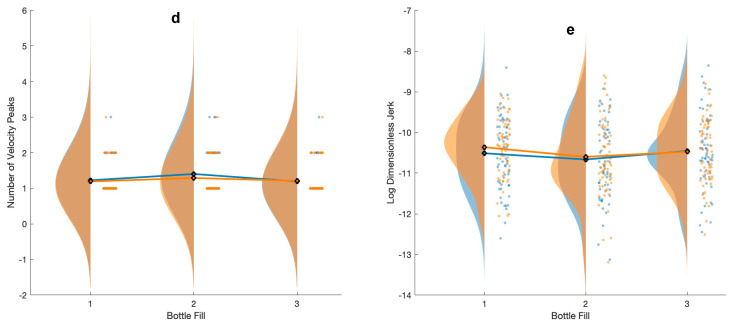
Raincloud plots [56] by hand and bottle fill (1 = empty, 2 = half, and 3 = full) for (**a**) average velocity, (**b**) peak velocity, (**c**) straightness ratio, (**d**) number of velocity peaks, and (**e**) log dimensionless jerk.

There was a main effect of bottle fill for average velocity and peak velocity, meaning that there was a significant change in average velocity and peak velocity at different bottle fill levels. A single subject example of the velocity curves across all bottle weights is provided in Figure 2 to demonstrate how the velocity of the movement varied between different bottle conditions. Fisher’s LSD post hoc analysis showed significantly slower average velocity (t(60) = 2.92, *p* = 0.005) and peak velocity (t(60) = 3.27, *p* = 0.001) when transporting the half-full bottle compared to the empty bottle. There was a main effect of hand for average velocity and peak velocity as well (Table 3 and Table 4; Figure 3), with the dominant hand overall showing slower average and peak velocities than the non-dominant hand. All other main effects and interaction effects did not show a significant difference.

## 4. Discussion

The aim of this study was to evaluate the effects of changing object weight on functional reach coordination in a reach-to-drink task in the context of DST. We evaluated changes in speed, straightness, and smoothness of the withdrawal movement (transporting the bottle to the mouth) in typically developing school-aged children. Three-dimensional motion capture data were collected for dominant and non-dominant hand transport of a bottle with three different weights based on fill level: empty, half-full, and full. Based on changes observed in our previous study, we hypothesized that increasing weight would result in slower, less straight, and less smooth withdrawal movements. These hypotheses were partially supported by our results, with slower average velocity and peak velocity with the half-filled bottle compared to the empty bottle but with no other significant differences. We also predicted that movements with the dominant hand would be faster, straighter, and smoother than the non-dominant hand; however, our results showed slower average velocity and peak velocity with the dominant hand and no difference in straightness and smoothness. Although not all our hypotheses were supported, the results are consistent with DST, representing a complex interplay between timing and coordination of hand movement, object weight (a task constraint), and handedness. Moreover, these results provide normative values for the kinematics of hand movement in a functional reach-to-drink task in typically developing school-aged children and will inform the individualization of intervention design for children with neuromotor disorders.

### 4.1. Object Constraints

There were significant differences in average and peak velocity of hand movement with different bottle fill levels. The average velocity of hand movement was an average 38.3 mm/s slower transporting the half-full bottle to the mouth compared to the empty bottle, while peak velocity was an average 73.6 mm/s slower. This partially supports our hypothesis, which is consistent with our previous study comparing reach-to-eat and reach-to-drink in typically developing school-aged children, and it is consistent with research in adults performing a reach-to-drink task with open cups with varying liquid levels [36,57]. These results also indicate a similar response to increased weight in withdrawal and prehension movements, as increased task difficulty for adults in previous studies resulted in slower movements [27,28,30,35]. Flindall et al. however, only found a significant decrease in peak velocity in the left hand with increased liquid level in the cup [27]. Based on DST, greater strength or motor control in the dominant or right hand might interplay differently with the changes in bottle weight.

Contrary to our hypotheses, there was no difference in straightness or smoothness between bottle weights. This is different than the changes observed in prehension movements in adults when task constraints are manipulated [35]. The average values of the straightness ratio and the number of velocity peaks for all bottle weights were very close to 1, which is potentially indicative of a ceiling effect. It is possible that the increase in weight was not large enough to cause a change in reach execution that would impact straightness or smoothness. It is also possible that straightness and smoothness are more affected in the visually guided prehension movement than they are for the somatosensory-guided withdrawal movement [35,53]. Perhaps speed is altered to maintain straightness and smoothness to prevent the possibility of the liquid sloshing. This might be indicative of a precision control effect.

### 4.2. Hand Dominance

We observed a main effect of slower average velocity and peak velocity by an average of 18.3 mm/s and 32.3 mm/s, respectively, with the dominant hand compared to the non-dominant hand during the withdrawal phase of the reach-to-drink task, which did not support our hypotheses [22,34,50]. In our previous study investigating the kinematics of the Reach and Grasp Cycle in school-aged children performing three different functional reaching tasks (reach-to-drink, reach-to-eat, and bilateral reach to mouth), we found no significant difference between dominant and non-dominant hands [36]. There are multiple possible explanations for this, as there were differences in both the task and object between the two studies, as our previous study used an open cup.

In support of our results, previous researchers have theorized that slower movement with the dominant hand than the non-dominant hand is the result of better precision control in the dominant hand [21]. Although we found no difference in straightness or smoothness to indicate better precision with the dominant hand, our results are similar to results of Nelson et al. in adults performing prehension movements [35]. They found that the dominant right hand moved slower than the non-dominant left hand when the grasping task was more difficult. In that study, they also found that the left-hand-dominant adults moved slower with their non-dominant right hand in the more difficult task. Other studies have also provided support for a right-hand advantage in adults for reach-to-grasp tasks due to a potential left hemisphere specialization in higher-level motor planning and processing of visuomotor information [27,58,59]. While we did not find any significant differences between right-hand- and left-hand-dominant individuals, with left-hand-dominant children making up only 16% of our sample, we cannot rule out the possibility of a right-hand advantage in this population.

Last, our results showed no significant difference in straightness and smoothness of hand movements with the dominant hand compared to the non-dominant hand. This is consistent with our previous study [36]. Additionally, previous comparisons between children with CP and typical development (without regards to hand dominance) using the Reach and Grasp Cycle showed no difference in straightness ratio or number of velocity peaks between the two groups [1,8]. Studies in adults have shown straighter and smoother prehension movements with the dominant hand compared to the non-dominant hands [35]. One possibility for our findings is that kinematics of withdrawal movements are differently affected by changes in object weight, potentially because it involves more motor control than motor planning and is controlled by different neural systems. Another possibility is that the change in weight in this study was not large enough to induce differences between dominant and non-dominant hand movements. It is also possible that our contradictory results to those found in adults are due to our participants being school-aged or differences in the type of task and grip used. 

### 4.3. Limitations and Future Directions

One limitation of this study was the need to use capped bottles and have participants simulate drinking from the bottles rather than drinking from an open cup. While this change made the task a little less ecologically valid as a functional task, this was a necessary safety precaution taken to prevent spread of infection during the COVID-19 global pandemic. This ultimately changed the constraints from a naturalistic reach-to-drink task, as the participants had no risk of spilling the contents. This might be why we saw no differences between the half-full and full bottle conditions. This is why we highlighted weight of the bottle as the main constraint.

A second limitation of this study is that the conditions were presented in the same order to every participant. This might have led to a practice effect with the full bottle condition, which might be why we found no significant difference in speed between the half-full and full bottle conditions. In the future, the order of trials should be randomized for each participant to mitigate the potential of a practice effect.

It is possible that using a transparent bottle provided visual cues for the participants, making the task easier, even though withdrawal movements are shown to be mostly somatosensorily guided. A future study could test this either using opaque bottles to remove visual cues or by tracking eye movement and blinks.

Lastly, we were unable to complete a valid comparison of left-hand vs. right-hand dominance and between age groups due to their unequal distribution in our sample. It is possible that there are significant differences based on these participant characteristics and that our sample was not sufficient to capture them. This is a potential avenue for future investigation that could help to increase our understanding of typical development in the execution of functional upper-extremity movements in a reach-to-drink task. In this future study, we will also apply a validated assessment to determine hand dominance in the participants.

To broaden our understanding of withdrawal movements in school-aged children, to best inform precise rehabilitation, future studies should focus on evaluating kinematic changes by varying different types of constraints, such as the shape and size of the cup or bottle. Additionally, to increase the utility of this task as a functional measure, future studies should evaluate test–retest reliability and responsiveness to change with intervention. Finally, separating analysis by age with a future, larger sample will allow for more precise normative values.

### 4.4. Clinical Implications

Increasing the weight of the bottle from empty to half-filled in a functional ADL reach-to-drink task caused slower average velocity and peak velocity of the withdrawal movement in this study. This indicates that heavier objects might trigger more precision-control mechanisms in the motor cortex than lighter objects. The weight of the object should be considered for therapeutic interventions for children with neuromotor disorders targeting precision and accuracy of withdrawal movements. Conversely, if the goal of the intervention is to increase speed of the movement, a lighter-weight object might be more appropriate for practice. Moreover, comparison of our results with reach-to-place tasks indicates that a reach-to-drink task might require more somatosensory feedback to control the withdrawal movement. Reach-to-drink tasks might be considered as a skill progression once a child is successful with a reach-to-place task.

Significant differences in end-point kinematics of average velocity and peak velocity were detected between the dominant and non-dominant hands in this study. This indicates that the same methods might also detect differences between the affected and unaffected upper extremity in children with neuromotor disorders. While further research must be carried out to confirm test–retest reliability and determine a minimally clinically important difference in these kinematic variables, our results provide preliminary support for the future application of these variables using 3D motion capture to track changes in upper-extremity timing and coordination in children with motor impairment and guide design of individualized rehabilitation.

## 5. Conclusions

This study showed that for typically developing school-aged children, a change in bottle weight that could be expected to occur over the course of eating a lunch is sufficient to alter their hand kinematics. Specifically, differences in speed in the withdrawal phase of a reach-to-drink task were detected with varying bottle weight and comparing the dominant and non-dominant hands. These results are important for increasing our understanding of the complex interplay of dynamic systems in human movement. This will help to inform the design of individualized treatment for upper-extremity rehabilitation targeting reach-and-grasp tasks for ADL participation for children with neuromotor disorders. These results also provide preliminary support for the application of end-point kinematics to assess changes in upper-extremity function and coordination in school-aged children.

## Figures and Tables

**Table 1:** Bottle size and weight by condition.

Condition	Bottle Size (Diameter × Height (cm))	Weight (g)
Empty	6.2 × 14	31.5
Half		187.9
Full		346.5

**Table 2:** Spatial and temporal variables of reach.

Variable	Units	Description	Calculation
Average Speed	mm/s	Average velocity during the withdrawal movement	Velocity was calculated at each position of the hand marker cluster using a 4-point central difference numerical differentiation, then averaged across the movement
Peak Velocity	mm/s	Peak velocity reached during the withdrawal movement	Velocity was calculated at each position of the hand marker cluster using a 4-point central difference numerical differentiation
Straightness Ratio	ratio	a value closer to one indicates a straighter movement	Hand path length/straight line distance from start to end of the movement
Number of Velocity Peaks	count	Fewer velocity peaks indicate a smoother movement	Number of velocity peaks in a movement, with a peak defined as a change > 100 mm/s
Log Dimensionless Jerk	no units	Measures smoothness of movement, with less negative values indicating a smoother movement	The negative natural logarithm of the squared absolute value of jerk multiplied with the trial duration to the power of three and divided by the squared peak velocity [55]

**Table 3:** Main Effects.

Effects	Bottle Fill			Hand			Bottle Fill * Hand		
Num DF	2			1			2		
Den DF	60			30			60		
**Variables**	**F**	**Sig**	**η^2^*_p_***	**F**	**Sig**	**η^2^*_p_***	**F**	**Sig**	**η^2^*_p_***
Average Velocity (mm/s)	4.58	0.01 *	0.13	5.80	0.02 *	0.28	0.01	0.99	0.0003
Peak Velocity (mm/s)	5.34	0.007 *	0.15	7.31	0.01 *	0.33	1.69	0.19	0.05
Straightness Ratio	0.73	0.48	0.02	0.01	0.93	0.001	0.66	0.52	0.02
Number of Velocity Peaks	3.00	0.06	0.09	0.88	0.36	0.06	0.67	0.51	0.02
Log Dimensionless Jerk	1.90	0.16	0.06	1.01	0.32	0.06	0.55	0.58	0.02

* Significant main effect (*p* < 0.05).

**Table 4:** Mean (SD) of kinematic variables by hand and bottle fill.

		Bottle Fill			
Variable	Hand	Empty	Half	Full	Average of Fills
Average Velocity (mm/s) ^1,2^	Dominant	540.4 (147.0)	503.6 (149.1)	531.5 (124.1)	525.2 (140.7) ^1^
	Non-dominant	560.3 (137.3)	520.6 (142.6)	549.4 (154.6)	543.5 (145.2) ^1^
	Average of Both Hands	550.4 (142.0) ^2^	512.1 (145.6) ^2^	540.5 (139.9)	
Peak Velocity (mm/s) ^1,2^	Dominant	885.5 (234.3)	829.0 (212.9)	877.9 (175.0)	864.1 (209.2) ^1^
	Non-dominant	947.0 (231.6)	856.4 (183.4)	886.2 (195.9)	896.5 (207.0) ^1^
	Average of Both Hands	916.3 (234.0) ^2^	842.7 (198.4) ^2^	882.0 (185.0)	
Straightness Ratio	Dominant	1.042 (0.035)	1.050 (0.049)	1.045 (0.038)	1.045 (0.041)
	Non-dominant	1.047 (0.046)	1.048 (0.052)	1.040 (0.040)	1.045 (0.046)
	Average of Both Hands	1.044 (0.041)	1.049 (0.050)	1.042 (0.039)	
Number of Velocity Peaks	Dominant	1.19 (0.43)	1.29 (0.52)	1.21 (0.45)	1.23 (0.47)
	Non-dominant	1.22 (0.45)	1.40 (0.58)	1.19 (0.43)	1.27 (0.50)
	Average of Both Hands	1.21 (0.44)	1.34 (0.55)	1.20 (0.44)	
Log Dimensionless Jerk	Dominant	−10.36 (0.72)	−10.60 (0.95)	−10.47 (0.80)	−10.48 (0.83)
	Non-dominant	−10.51 (0.87)	−10.66 (0.92)	−10.45 (0.89)	−10.54 (0.90)
	Average of Both Hands	−10.41 (0.80)	−10.63 (0.93)	−10.46 (0.84)	

^1^ Significant main effect of hand, *p* < 0.05. ^2^ Significant difference between empty and half bottle with post hoc testing, *p* < 0.01.

## Data Availability

The raw data supporting the conclusions of this article will be made available by the authors on request.
